# When Safety Fails to Update: Altered Dopamine Prediction Signals in Extinction-Deficient Mice

**DOI:** 10.1523/ENEURO.0426-25.2025

**Published:** 2026-01-22

**Authors:** Mahmoud Kh. Hanafy

**Affiliations:** Department of Anatomy and Cell Biology, Schulich School of Medicine and Dentistry, University of Western Ontario, London, Ontario N6A 5C1, Canada

Fear extinction, the gradual reduction of conditioned fear in response to a previous threatening experience that no longer predicts danger, plays a crucial role in exposure-based therapies for anxiety and trauma-related disorders. Despite the efficacy of these therapies for many, a significant number of individuals encounter difficulties in extinguishing fear, resulting in treatment resistance. Understanding the mechanisms behind the variation in extinction success among individuals remains a central pursuit in neuroscience.

Recent studies have identified dopamine (DA) neurons within the ventral tegmental area (VTA) to influence the extinction process. In addition to their well-established role in signaling reward prediction errors, specific populations of VTA DA neurons also respond to the unexpected omission of an aversive stimulus, producing a signal indicative of safety. In animals capable of successful extinction, this burst of activity associated with stimulus omission diminishes over time, signaling the cessation of threat. However, whether this dynamic is altered in extinction-deficient models has not been extensively explored.

In their article “Altered dopamine signaling in extinction-deficient mice” (eNeuro>, 2025), the authors address this important question utilizing the 129S1/SvImJ (S1) strain, which serves as a model for impaired fear extinction (Gunduz-Cinar et al., 2025). By backcrossing DAT-Cre mice with the S1 mice and employing a combination of behavioral analyses, in vivo fiber photometry, and pathway-specific optogenetics, they reveal a selective disruption in extinction-related dynamics of VTA DA signaling that was not attributed to neuronal loss. Furthermore, attempts to artificially activate VTA DA neurons failed to improve behavioral outcomes, suggesting that the dysfunction observed pertains to the qualitative aspect of prediction-error coding rather than a basic quantitative deficit.

In their findings, the S1-DAT-Cre mice displayed normal acquisition of fear but exhibited a failure to reduce freezing behavior across extinction sessions, mirroring the extinction deficits found in the parental S1 line. Immunohistochemical analyses of tyrosine-hydroxylase-positive cells indicated no significant difference in the number of VTA DA neurons when compared with C57BL/6 (BL6) control mice. This observation shows that the deficits in extinction behavior originate from altered neuronal function rather than the loss of dopaminergic neurons.

Fiber photometry recordings demonstrated increased calcium activity in VTA DA neurons during the omission of an unconditioned stimulus (US) in both strains during early extinction sessions, indicative of a positive prediction-error-like signal (Salinas-Hernandez et al., 2018; Cai et al., 2020). Notably, in BL6-DAT-Cre mice, this response diminished by the time of extinction retrieval, aligning with successful fear extinction (Salinas-Hernandez et al., 2018). Conversely, S1-DAT-Cre mice maintained abnormally elevated omission-related activity throughout both extinction and retrieval phases, correlating with persistent freezing behavior. Thus, the extinction deficit observed in these mice reflects not a loss of dopaminergic signaling but an aberrant persistence of this activity, suggesting a dysregulation in the temporal dynamics of dopamine-mediated prediction error processing.

The authors further explored whether enhancing DA activity during the omission period could compensate for the signaling dysfunction. Using optogenetics to stimulate either VTA DA cell bodies or DA axons projecting to the infralimbic cortex during periods of omission protocols known to promote extinction learning in competent animals, they found no improvement in the extinction behavior of S1-DAT-Cre mice (Gunduz-Cinar et al., 2025). These negative outcomes are insightful as they indicate that the failures observed in this model cannot be remedied simply through indiscriminate dopaminergic activation. The underlying issue is likely due to mis-timed or projection-specific signaling or possibly to a disrupted distribution of aversive versus appetitive subpopulations of DA neurons. Additionally, previous research has indicated that certain VTA to infralimbic DA projections convey aversive information, suggesting that global activation could inadvertently activate competing pathways.

This study builds upon an extensive body of literature that implicates dopamine in extinction processes across multiple species (Singewald and Holmes, 2019). Polymorphisms in dopamine-related genes such as DAT, DRD3, DRD4, and COMT have been linked to impaired extinction and the severity of post-traumatic stress disorder (PTSD) symptoms. Pharmacological interventions that enhance dopaminergic transmission, including methylphenidate and ʟ-DOPA, have been shown to facilitate extinction memory in both rodent and human models (Whittle et al., 2016), so the effect in S1 mice appears to be transient. The findings reported herein clarify that although dopaminergic tone is supportive of extinction, the manner in which dopaminergic prediction-error signaling occurs is critically important.

Moreover, the inability to scale back VTA DA activity at safety cue offset in S1-DAT-Cre mice reflects observations in clinical settings, where PTSD patients display continued ventral striatal activation to safety cues despite undergoing repeated exposure therapy (Gerlicher et al., 2018). This suggests that a rigid or poorly calibrated dopamine prediction-error system may contribute to the resistance observed in treatments aimed at reducing fear responses.

Future investigations should focus on the cellular mechanisms underlying this persistent omission response. For instance, do the S1 mice differ in the proportions of VTA DA neurons that encode aversive versus rewarding stimuli? And are the inhibitory or feedback inputs that normally attenuate VTA activity dysfunctional? Moreover, recording or manipulating discrete populations of VTA to nucleus accumbens projections, where omission-related dopaminergic release promotes extinction in other models, could elucidate alternative targets for potential therapeutic interventions.

It is also critical that future studies include female mice and verify the efficacy of dopaminergic release during optogenetic interventions. These refinements will help discern whether the observed abnormal signaling results from intrinsic plasticity within the midbrain, defective feedback mechanisms from the prefrontal cortex, or inadequate downstream decoding.

By identifying altered VTA DA prediction-error dynamics as a key circuit correlate of extinction failure, this research highlights the connection between fundamental dopaminergic computations and clinically relevant phenomena such as treatment resistance. Strategies aimed at restoring appropriate timing and modulation of dopaminergic prediction-error signals, rather than simply amplifying dopamine levels, may ultimately enhance the effectiveness of exposure-based therapeutic approaches for anxiety and trauma-related disorders.
